# Development and Validation of a Nomogram for Predicting Nutritional Risk Based on Frailty Scores in Older Stroke Patients

**DOI:** 10.1007/s40520-023-02689-0

**Published:** 2024-05-18

**Authors:** Lei Liu, Chunyu He, Jiaxin Yang, Wenbo Chen, Yan Xie, Xiaofang Chen

**Affiliations:** Chengdu Medical College, Chengdu, 610083 Sichuan China

**Keywords:** Stroke, Elderly people, Frailty, Nutritional risk, Nomogram, Primary care

## Abstract

**Background:**

In older stroke patients with frailty, nutritional deficiencies can amplify their susceptibility, delay recovery, and deteriorate prognosis. A precise predictive model is crucial to assess their nutritional risk, enabling targeted interventions for improved clinical outcomes.

**Objective:**

To develop and externally validate a nutritional risk prediction model integrating general demographics, physical parameters, psychological indicators, and biochemical markers. The aim is to facilitate the early identification of older stroke patients requiring nutritional intervention.

**Methods:**

This was a multicenter cross-sectional study. A total of 570 stroke patients were included, 434 as the modeling set and 136 as the external validation set. The least absolute shrinkage selection operator (LASSO) regression analysis was used to select the predictor variables. Internal validation was performed using Bootstrap resampling (1000 iterations). The nomogram was constructed based on the results of logistic regression. The performance assessment relied on the receiver operating characteristic curve (ROC), Hosmer–-Lemeshow test, calibration curves, Brier score, and decision curve analysis (DCA).

**Results:**

The predictive nomogram encompassed seven pivotal variables: Activities of Daily Living (ADL), NIHSS score, diabetes, Body Mass Index (BMI), grip strength, serum albumin levels, and depression. Together, these variables comprehensively evaluate the overall health and nutritional status of elderly stroke patients, facilitating accurate assessment of their nutritional risk. The model exhibited excellent accuracy in both the development and external validation sets, evidenced by AUC values of 0.934 and 0.887, respectively. Such performance highlights its efficacy in pinpointing elderly stroke patients who require nutritional intervention. Moreover, the model showed robust goodness of fit and practical applicability, providing essential clinical insights to improve recovery and prognosis for patients prone to malnutrition.

**Conclusions:**

Elderly individuals recovering from stroke often experience significant nutritional deficiencies. The nomogram we devised accurately assesses this risk by combining physiological, psychological, and biochemical metrics. It equips healthcare providers with the means to actively screen for and manage the nutritional care of these patients. This tool is instrumental in swiftly identifying those in urgent need of targeted nutritional support, which is essential for optimizing their recovery and managing their nutrition more effectively.

**Supplementary Information:**

The online version contains supplementary material available at 10.1007/s40520-023-02689-0.

## Introduction

Cerebrovascular events, commonly known as strokes, are characterized by acute neurological deficits due to localized or generalized cerebral tissue ischemia or hemorrhage. These neurological disruptions carry significant clinical implications for the affected individuals [1]. Statistics from the World Health Organization reveal that strokes impact approximately 15 million people worldwide every year, with a sobering 5.8 million resulting in death. These figures position strokes as the leading cause of disability and the second primary cause of death globally [2]. There is an undeniable escalation in the prevalence and associated disabilities of strokes, most saliently among the geriatric population—defined in this context as individuals aged 60 years and older. This upsurge detrimentally bears upon their life expectancy and overall quality of life. A nexus of physiological deficits, augmented disease severity, and psychological afflictions often fosters reduced resilience and augmented susceptibilities in stroke survivors, frequently converging to a state of frailty. The ramifications of this state are particularly trenchant among the old, critically impeding their functional rehabilitation and overall well-being [3]. In gerontological paradigms, frailty is emblematic of a marked functional regression, and it is inextricably associated with a spectrum of detrimental sequels. These encompass instances like inadvertent falls, functional debilitations, recurrent hospital readmissions, and an elevated risk of mortality [4]. Recent research insights delineate a significant correlation between frailty and nutritional status. In older adults, nutritional risk factors are closely associated with the decline in muscle mass and strength, directly influencing the onset and progression of frailty. This muscle loss significantly impacts their physical functionality, raising the risk of falls and contributing to other health challenges such as depression and cognitive decline. Aznar-Tortonda et al. emphasize the need for early detection of frailty, which can identified by physical parameters like muscle strength, a key aspect highlighted in the FRAIL scale. This connection between muscle loss and frailty underscores the importance of monitoring nutritional health to prevent frailty in older adults [5, 6]. These factors also serve as risk determinants for the onset of frailty. Patients exhibiting frailty often present with pronounced malnutrition, which, in turn, can exacerbate the frail condition, creating a vicious cycle [7]. Consequently, the prognostication and intervention pertaining to nutritional risks in frail individuals are of paramount importance. Such strategies not only ameliorate the nutritional quality of life of patients but also impede the progression of frailty, diminishing the likelihood of unfavorable outcomes. At present, various nutritional risk assessment instruments—including the Subjective Global Assessment (SGA), Nutritional Risk Screening 2002 (NRS 2002), and Malnutrition Universal Screening Tool (MUST)—have gained widespread adoption [8, 9]. However, the specificity and sensitivity of these tools in catering to the nuanced characteristics and requisites of frail older stroke survivors, particularly those inhabiting the Asia-Pacific region, remain contentious. Therefore, devising a nutritional risk predictive model tailored specifically for frail older stroke patients holds paramount clinical significance. By adeptly identifying individuals within this high-risk bracket, such a model can substantially enhance their clinical management and prognosis, offering both pragmatic implications and scholarly merit.

## Materials and methods

### Participants

Using a convenience sampling method, we selected stroke inpatients from Class A tertiary hospital (It is a medical institution classified in accordance with the provisions of China's current "Hospital Classification and Management Measures", and is the highest level in the "three levels and six grades" classification of hospitals in mainland China.) in Chengdu who met the inclusion and exclusion criteria. We favored this sampling method for its efficiency in recruiting a specific patient population amidst resource constraints, offering a more direct and time-efficient approach compared to other methods.

The inclusion criteria included: (1) aged ≥ 60 years, meeting the diagnostic criteria for cerebrovascular disease from the Fourth National Conference [10], diagnosed by CT, and have a confirmed stroke diagnosis via MRI scans; (2) we reviewed the patient’s medical records and consulted with physicians to confirm that the patients did not have aphasia or dementia, could communicate effectively, and were willing to participate in the study; (3) FRAIL scale score ≥1; and (4) informed and voluntarily willing to participate in the study (Written informed consent was obtained from the patients themselves. In situations where the patient was unable to sign due to physical or cognitive limitations, their direct relatives provided consent, ensuring the patient's understanding of the study).

The exclusion criteria included: (1) patients with transient ischemic attack; (2) patients who underwent emergency surgery or received thrombolytic therapy; (3) presence of other severe comorbidities including but not limited to malignant tumors, significant hepatic or renal dysfunction, major trauma, respiratory failure, severe arrhythmia, frequent angina, heart failure, and myocardial infarction; (4) those who received enteral or parenteral nutritional support before their hospital admission; and (5) history of severe psychiatric symptoms.

Questionnaires that contained clear logical errors, had missing scale content, or exhibited outliers after data processing would be excluded.

### Sample Size

Based on the predictive model sample size calculation proposed by Riley et al. [11], the study required a sample size ranging from 378 to 423 cases. Accounting for a potential 10% of ineffective cases, the minimum sample size was set at 416. The actual study included 434 cases. For a detailed description of the sample size calculation formula and method, see Supplementary Method S1.

### Variable Selection

Predictive variables for the nutritional risk of stroke patients were identified through a literature review, group discussions, and consultations with experts in the fields of neurology and nutrition. The final set of variables selected for collection included: (1) Demographics: sex, age, educational level, marital status, family monthly income, stroke type, comorbidity of chronic diseases, consumption of ≥3 prescription medications, history of smoking, alcohol consumption, and falls. (2) Laboratory indicators such as albumin, total protein, triglycerides, cholesterol, and hemoglobin. These were sourced from the patient's medical records and the laboratory test results from the day of the assessment. If no tests were conducted on the assessment day, the most recent laboratory results prior to the assessment date were used. (3) Body Mass Index (BMI) calculation, measurements of upper arm circumference, triceps skinfold thickness, calf circumference, waist circumference, hip circumference, and mid-upper arm muscle circumference. (4) Utilized the National Institutes of Health Stroke Scale (NIHSS) which consists of 15 items, with a total score ranging from 0 to 42. Scores are categorized as: 0–1 divided into normal or near-normal, 2–4 in mild impairment, 5–15 in moderate impairment, and >15 in severe impairment. (5) The Barthel index is used to assess the ADL of patients. Includes 10 aspects, non-dependence 100 points, mild dependence 61 to 99 points, moderate dependence 41 to 60 points, and gravity dependence ≤40 points. (6) Depression is assessed using the Geriatric Depression Scale (GDS-15). It consists of 15 items with a maximum score of 15. Scores are interpreted as: 0 to 4 for normal, ≥5 indicative of depression. A higher score indicates more severe depression symptoms.

### Frailty assessment

The FRAIL scale, endorsed by experts in care for older individuals from the International Association of Nutrition and Aging (IANA) and grounded in established frailty criteria and indices [12], consists of five straightforward self-reported questions addressing fatigue, resistance reduction, ambulation, illness, and weight loss. The scale totals a maximum of 5 points: scores of ≥3 indicate frailty, 1–2 suggest pre-frailty, and a score of 0 represents robustness or non-frailty.

### Nutritional Risk Definition

The NRS-2002 is recommended by the European Society for Parenteral and Enteral Nutrition (ESPEN) [13] and is used to assess nutritional risks in adult inpatients. This tool has been proven to possess strong validity and reliability in prospectively gauging shifts in a patient's nutritional status. The scale consists of three distinct segments: the degree of influence of disease on nutritional status (up to 3 points), the level of malnutrition (comprehensive evaluation of changes in weight over the past 3 months, changes in dietary intake over the last 1 week and BMI values, up to 3 scores) and the age score (at 1 point for the age of ≥70 years). Cumulatively, these scores have a maximum total of 7. A score of ≥3 implies that a patient is at nutritional risk, while scores below 3 signify no immediate nutritional peril.

## Data Collection Methodology

This study received ethical approval from the Ethics Committee of Chengdu Medical College (Approval number: 2022NO.22) and adheres to the principles set out in the Helsinki Declaration. Prior to participation, all participants furnished informed consent. General demographic details and the aforementioned scores were gathered on the day of admission, while laboratory indicators were extracted directly from the patient's medical records. To uphold the integrity of the data, trained investigators administered one-on-one, on-site questionnaire surveys. In situations where patients were unable to provide answers independently, their spouses or legal guardians were interviewed instead.

## Data Preprocessing

Variables with missing values were removed to ensure data integrity. Outliers were identified using the Interquartile Range (IQR) method, and data entries with illogical anomalies were discarded. Such outliers typically arise from input errors or measurement inconsistencies and deviate significantly from the overall data distribution. To maintain the authenticity of the dataset while adjusting for unit differences, the data were standardized. For clinical applicability, BMI was transformed into a binary classification, but all other data remained in its original format.

## Statistical Analysis

Data were input into Excel and then double-checked for accuracy, with corrections made as required. Data analysis was conducted using IBM SPSS Statistics 26.0 (IBM Corp., Armonk, NY) and R version 4.3.0 (R Foundation for Statistical Computing, Vienna, Austria). The specific methodologies employed included: (1) Count data were represented using rates and composition ratios. Measurement data that followed a normal distribution were described using the mean ± standard deviation. Non-normally distributed data were represented using the median and interquartile range. (2) The Chi-squared test and Fisher's exact test were applied to assess distribution differences for categorical variables. Ranked data differences between groups were analyzed using the Wilcoxon rank-sum test. The Mann–Whitney U test was used for non-normally distributed data. A *p*-value less than 0.05 was deemed statistically significant. (3) Preliminary factor screening for predictions was done using LASSO regression. The optimal penalty coefficient for the model was chosen based on the lambda value that corresponded to the cross-validated error within one standard deviation of the minimum error. Following this, a binary logistic regression analysis of variables filtered by the LASSO regression was conducted to discern the final predictors and construct a nomogram model; (4) Model discrimination was assessed with the Bootstrap-based Area Under the Curve (AUC). Model calibration was examined through the Hosmer–Lemeshow goodness-of-fit test, calibration plots, and the Brier score. The model's clinical utility was further evaluated using a Decision Curve Analysis (DCA) conducted with the rmda package in R.

## Results

### 1Participant Characteristics

A total of 570 patients were included in this study. Of these, 434 were allocated to the development set, while 136 served as the external validation set. The incidence of nutritional risk was 49.5% in the development set and 51.4% in the external validation set. The average age of patients in the development set was 72.03 years, with an age range of 60 to 90 years. For the external validation set, the average age was 70.53 years, spanning an age range of 60 to 89 years. The basic characteristics and univariate analysis results of the modeling set are presented in Table 1. Details pertaining to the external validation set can be found in Supplementary material Table S1.

**Table1:** Baseline characteristics between stroke patients with different nutritional risk groups in the development sets

Characteristics	Group			*χ*^*2*^/*Z*/*T*	*P* Value
	Overall (n = 434)	Nutritional risk (n = 215)	Non-nutritional risk (n = 219)		
Age				−5.967	<0.001
60 ~ 70	176(40.6)	63(29.3)	113(51.6)		
70 ~ 80	161(37.1)	82(38.1)	79(36.1)		
80 ~ 90	97(22.4)	70(32.6)	27(12.3)		
Sex				1.583	0.208
Male	214(49.3)	107(47.8)	117(52.2)		
Female	220(50.7)	113(53.8)	97(46.2)		
Education				−3.491	<0.001
Primary school and below	249(57.4)	138(64.2)	111(50.7)		
Junior high	105(24.2)	53(24.7)	52(23.7)		
High school	75(17.3)	23(10.7)	52(23.7)		
College and above	5(1.2)	1(0.5)	4(1.8)		
Income				−4.011	<0.001
<2000	197(45.4)	114(53.0)	83(37.9)		
2000~4000	170(39.2)	83(38.6)	87(39.7)		
≥4000	67(15.4)	18(8.4)	49(22.4)		
Smoking history	107(24.7)	67(31.2)	40(18.3)	9.716	0.002
Drinking history	96(22.1)	62(28.8)	34(15.5)	11.16	<0.001
Physical exercise				−4.019	<0.001
Non-exercise	108(24.9)	72(33.5)	36(16.4)		
Sometimes exercise	172(39.6)	81(37.7)	91(41.6)		
Regular exercise	154(35.5)	62(28.8)	92(42.0)		
Taking ≥ 3 prescription drugs per day	164(37.8)	101(47)	63(28.8)	15.303	<0.001
Fall history (within one year)	104(24.0)	61(29)	43(19.2)	5.773	0.016
ADL				−14.366	<0.001
Independent	183(42.2)	19(8.8)	164(74.9)		
Mildly dependent	101(23.3)	61(28.4)	40(18.3)		
Moderate dependent	100(23.0)	90(41.9)	10(4.6)		
Heavy dependent	50(11.5)	45(20.9)	5(2.3)		
First time	284(65.4)	129(60)	155(70.8)	5.571	0.018
Disease course				−2.783	0.005
<1	296(68.2)	132(61.4)	164(74.9)		
1~5	95(21.9)	60(27.9)	35(16)		
≥5	43(9.9)	23(10.7)	20(9.1)		
NIHSS score				−9.637	<0.001
0~1	106(24.4)	14(6.5)	92(86.0)		
1~4	168(38.7)	82(38.1)	86(39.3)		
5~15	160(36.9)	119(55.3)	41(18.7)		
Hypertension	212(48.8)	107(51.0)	105(46.9)	0.721	0.396
Diabetes	136(31.3)	103(47.9)	33(15.1)	54.373	<0.001
BMI				−7.256	<0.001
<18.5	53(12.2)	48(22.3)	5(2.3)		
18.5~23.9	277(63.8)	139(64.7)	138(63)		
24~27.9	104(24.0)	28(13)	76(34.7)		
Upper arm circumference	25(23.5, 26.075)	24(22.5, 25)	25.5(24.5, 27)	−7.78	<0.001
Triceps skinfold thickness	11.25(9.0, 15.0)	11(8, 14)	12(10, 15.875)	−4.275	<0.001
Waist-hip ratio	0.941 ± 0.029	0.934 ± 0.019	0.948 ± 0.019	−5.072	<0.001
Upper arm muscle circumference	20.98(19.6, 22.55)	20.3(18.98, 21.83)	21.6(20.29, 22.86)	−5.921	<0.001
Calf circumference <31 cm	233(53.7)	163(75.8)	70(32)	−9.074	<0.001
Grip	15.25(7.8, 21.0)	9.2(5.4, 15.2)	19(15, 22.8)	−10.908	<0.001
Total cholesterol	4.23(3.43, 4.91)	3.92(3.14, 4.59)	4.58(3.82, 5.27)	−6.408	<0.001
Albumin	40.1(35.6, 43.83)	35.89(33.7, 39)	43.1(40.68, 45.48)	−13.405	<0.001
Total protein	66.75(60.5, 72.72)	61.6(57.3, 66.6)	70.9(67.0, 74.8)	−11.337	<0.001
Triglyceride	1.26(0.98, 1.74)	1.11(0.87, 1.48)	1.44(1.08, 2.06)	−6.105	<0.001
Hemoglobin	128.5(115, 142)	119(108, 134)	135(124, 147)	−7.549	<0.001
Depression	174(40.1)	156(72.6)	18(8.2)	−13.658	<0.001
CRP	2.0(0.7, 10.0)	7.2(2.0, 15.0)	1.0(0, 3.0)	−10.482	<0.001

### Variable Selection

**Fig. 1:**
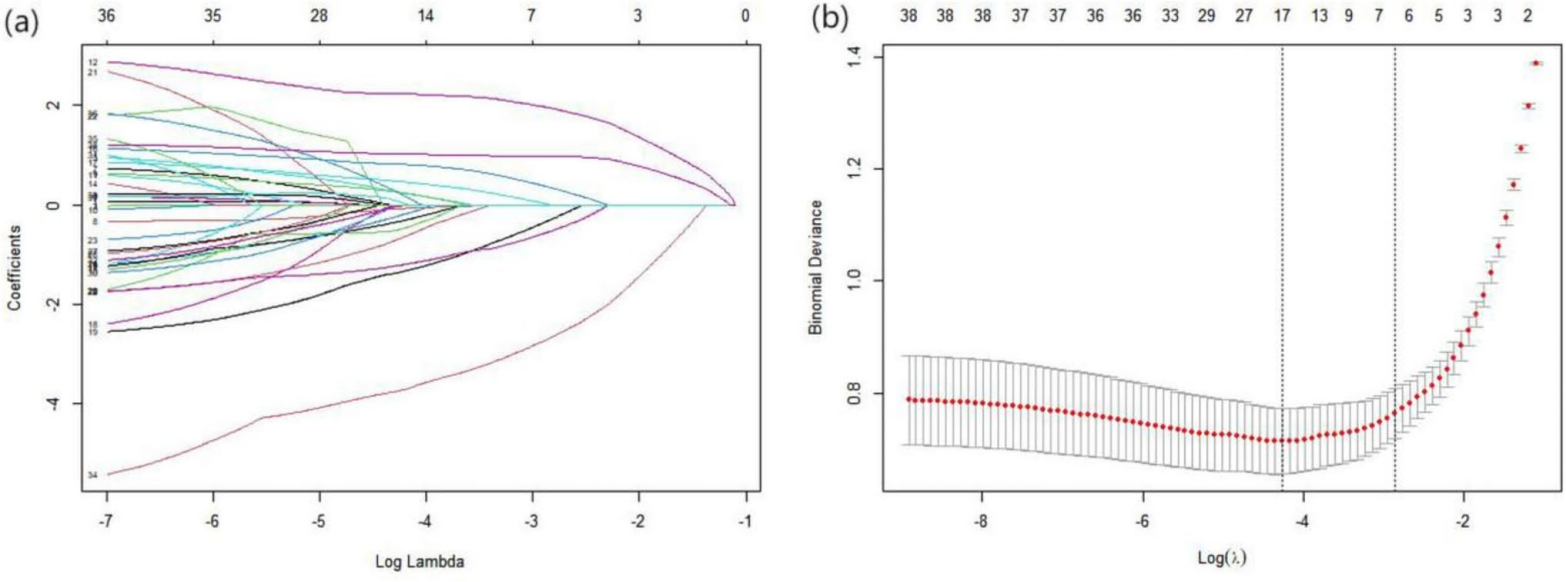
LASSO regression screened 7 potential out of 38 candidate variables. Note: (a) Displays the distribution of LASSO regression coefficients for 38 characteristics, producing a logarithm (lambda) sequence coefficient profile graph; (b) Two vertical dashed lines from left to right represent lambda.min and lambda.1se, respectively. Lambda.min corresponds to the λ value that results in the smallest estimated model error, while lambda.1se corresponds to the λ value where the internal cross-validation error is at its maximum within one standard deviation. By determining the optimal λ, seven non-zero coefficients are derived

In this analysis, nutritional risk presence served as the dependent variable. Statistically significant factors identified from the univariate analysis were integrated into the LASSO regression. A tenfold cross-validation, along with minimization criteria, was employed to ascertain the optimal coefficient λ (Fig. 1a, b). The outcomes revealed that at a λ value of 0.056, seven variables were discerned with non-zero coefficients. These encompassed daily living activity capability, NIHSS score, diabetes, BMI, grip strength, serum albumin, and depression. The importance ranking of these variables can be found in Supplementary Figure S1 in the supplementary materials.

### Model Development

**Fig. 2:**
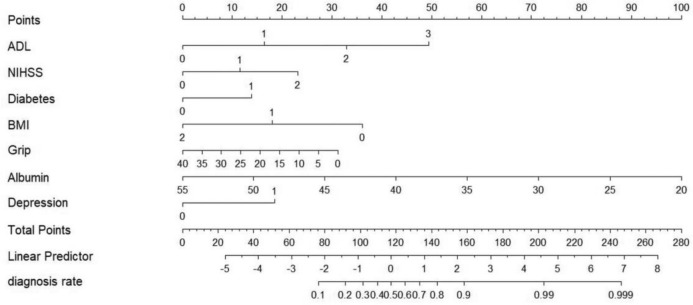
Nomogram for predicting nutritional risk in frail older stroke patients

Based on the seven pivotal variables identified through the LASSO regression, and using the presence of nutritional risk as the outcome event, logistic regression was employed (Table 2). In utilizing this nomogram (Fig. 2), scores associated with each predictive variable are combined to derive a comprehensive score. This consolidated score can then be used to estimate the likelihood of a patient having nutritional risk. To illustrate, consider a hypothetical patient with a moderate dependency in ADL (accruing 32 points), an NIHSS score of 7 (22 points), presence of diabetes (11 points), a BMI of 18.1 kg/m^2^ (38 points), grip strength measuring 15 kg (20 points), serum albumin concentration of 35 g/dL (58 points), and absence of depression (0 points). The patient's aggregate score would be roughly 181 points, corresponding to a 95% chance of experiencing nutritional risks.

**Table 2:** Results of binary logistic regression analysis

variant	*β*	*SE*	Waldχ^2^	*P*	*OR*	95% *CI*
Constant	5.747	1.793	10.273	0.001		
ADL	0.883	0.212	17.435	<0.001	2.419	1.598–3.662
NIHSS	0.619	0.224	7.618	0.006	1.857	1.197–2.883
Diabetes	0.741	0.349	4.504	0.034	2.098	1.058–4.160
BMI	-0.969	0.309	9.820	0.002	0.380	0.207–0.696
Grip	-0.042	0.023	3.398	0.065	0.959	0.917–1.003
Albumin	-0.153	0.040	14.515	<0.001	0.858	0.793–0.928
Depression	0.991	0.401	6.121	0.013	2.694	1.229–5.909

### Internal and External Model Validation

**Fig. 3:**
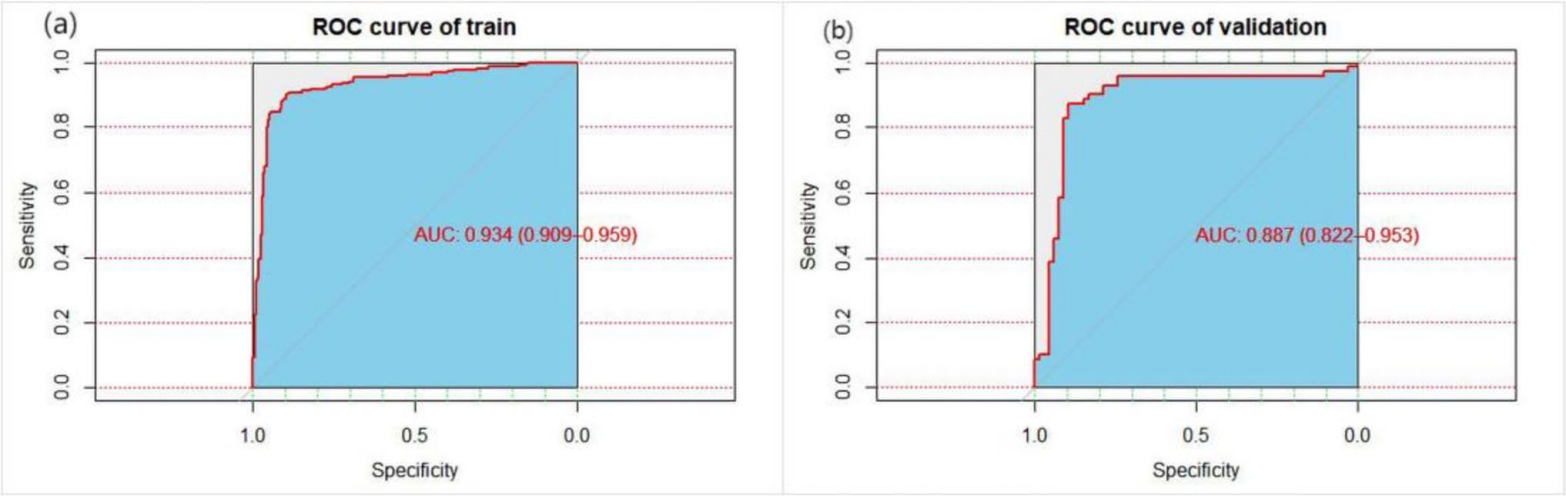
Roc curve of nutrition risk model in training and validating cohorts

**Fig. 4:**
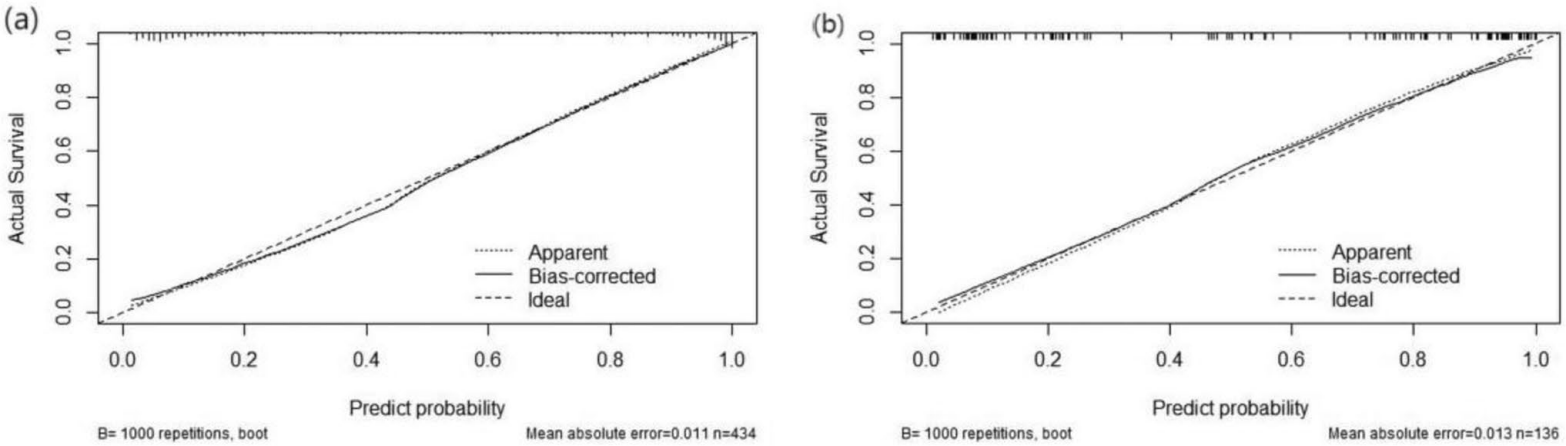
Calibration curve of nutrition risk model in training and validating cohorts

**Fig. 5:**
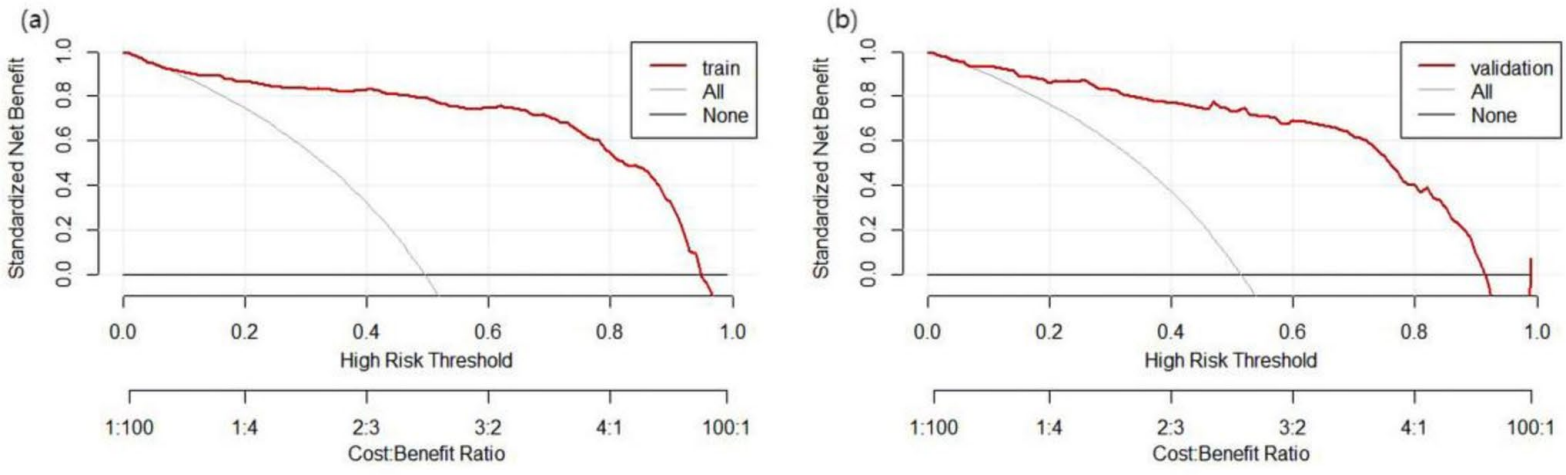
DCA curve of nutrition risk predictive nomogram in training and validating cohorts

Using R 4.3.0, the ROC curve for the predictive model was generated. Bootstrap resampling was executed with 1,000 iterations for internal validation of the model. The post-calibration AUC was determined to be 0.934 (95% CI: 0.909 to 0.959) as depicted in Fig. 3a, indicating a good distinction capability of the model. The model's accuracy in the development set was recorded as 89.17%, with a specificity of 0.874. With a Brier score of 0.088 and complemented by the calibration plot (Fig. 4a) and the Hosmer–Lemeshow test (χ^2^ = 12.398, P = 0.13), the model's predictive precision was evident. Furthermore, the Decision Curve Analysis (DCA) demonstrated in Fig. 5a revealed that the model's net benefit surpassed that of "treat all" or "treat none" strategies.

For external validation, 136 stroke patients seen between May and July 2023 were utilized. The AUC of the model was 0.887 (95% CI 0.822 to 0.953) and is presented in Fig. 3b. A Brier score of 0.106 was noted, and the results of the Hosmer–Lemeshow test were χ^2^ = 13.634, P = 0.092. The accuracy and specificity of the model were 86.76% and 0.914, respectively, indicating that the model performed well in its predictive efficacy in the external validation set. Both the calibration curve (Fig. 4b) and DCA (Fig. 5b) further affirmed the model's discriminating ability and clinical utility.

## Discussion

### Elevated Nutritional Risk in older Stroke Patients with Frailty

Nutritional risk goes beyond merely observing the occurrence of malnutrition. It is characterized by current or prospective nutrition-related factors that might predispose a patient to clinical adversities. Unlike malnutrition, nutritional risks underscore the clinical implications of undernourishment, exhibiting a higher prevalence than mere malnutrition itself [14]. Research indicates that the prevalence of malnutrition and nutritional risks in stroke patients can vary significantly, influenced by factors such as sample characteristics, regional differences, and assessment methodologies. Cui et al. reported that the incidence of malnutrition among hospitalized patients with neurological disorders is approximately 10%, while the proportion at nutritional risk is around 20% [15]. In contrast, studies employing the Global Leadership Initiative on Malnutrition (GLIM) criteria have documented higher malnutrition rates, with 43% reported by Sato K et al. [16], and 35.2% by Kobayashi D et al. [17]. Additionally, Nozoe M et al., using the Geriatric Nutritional Risk Index (GNRI) to evaluate the nutritional status of elderly stroke patients, found a 13.0% incidence of malnutrition risk [18]. This study discerned a nutritional risk incidence rate of 49.5% in older stroke patients exhibiting frailty, and 51.5% in the external validation cohort. These figures markedly surpass prior findings, insinuating that stroke patients with functional impairments confront more adverse nutritional statuses.

The literature emphasizes that malnutrition and frailty often coexist in hospitalized geriatric stroke patients [19, 20]. This concomitance not only perpetuates each condition but also culminates in a detrimental feedback loop, posing amplified health risks. Such mutual exacerbation might stem from shared pathophysiological underpinnings, with both conditions impinging upon seniors' autonomy, life quality, and healthcare expenditure. Frailty, typified by a decline in functional capacity, often manifests as weight loss, muscle atrophy, and diminished appetite. Concurrently, strokes can induce metabolic alterations in patients. Therefore, the concurrence of frailty with stroke elevates the predisposition to nutritional risks. Elevated nutritional risks might exacerbate age-associated muscle atrophy and diminished strength, potentially leading to functional incapacitation, mood disorders, falls, compromised immune response, and cognitive impairments, thereby intensifying frailty [21].

Both the attenuated phenotype model and the holistic frailty conceptual model integrate nutrition as a pivotal explanatory factor. Frailty undergoes a dynamic evolutionary trajectory. Timely recognition and intervention can potentially decelerate the progression of frailty. Given that nutrition remains a modifiable determinant in the trajectory of frailty development, the early identification and management of nutritional risks in frail older stroke patients are paramount. Such interventions not only enhance patient recovery and mitigate complications but also augment patients' quality of life.

### Analysis of Pertinent Risk Factors

This study established a model encompassing ADL, NIHSS scores, diabetes, BMI, grip strength, serum albumin levels, and depression to forecast the nutritional risk in frail older stroke patients. Previous research has demonstrated a bidirectional relationship between ADL and nutrition, while the ability to perform daily activities can predict nutritional status, nutrition which, in turn, significantly influences one's daily functioning. A compromised ability to conduct daily tasks might signify challenges related to feeding and other activities, resulting in inadequate nutritional intake and potential deterioration of a patient's nutritional status [22, 23]. Furthermore, reduced motor function can impact muscle mass and metabolic rate, elevating nutritional risk.

NIHSS scores serve as indicators of neural damage severity. Elevated scores suggest pronounced neurological deficits, which might compromise eating abilities due to issues like mastication and dysphagia. Severe cases often coincide with inflammatory responses and metabolic disturbances, exacerbating nutritional inadequacies [24].

Many stroke patients concurrently present with diabetes, potentially stemming from the stroke's impact on neuroendocrine systems. This can diminish insulin sensitivity and elevate insulin resistance risk [25]. Insulin resistance can attenuate cellular insulin responsiveness, interfere with protein synthesis and degradation, and amplify nutritional risk. Concurrently, it jeopardizes peripheral systems by promoting free fatty acid circulation and deposition into myocytes, leading to intramuscular fat accumulation and perturbed muscle metabolism [26].

Physiologically, metrics like BMI and grip strength correlate with nutritional risk, reflecting body fat, muscle mass, and muscular strength—essential parameters for nutritional evaluation [27]. Post-stroke muscle and weight loss are frailty hallmarks and diminished BMI and grip strength might signify muscle and adipose tissue loss. These phenomena might be underpinned by metabolic disorders, inflammatory responses, and reduced motor function, exacerbating nutritional inadequacies [28].

Serum albumin, a standard nutritional marker, encapsulates a patient's nutritional health and protein metabolism dynamics. Protein deficits in stroke patients, often manifesting as hypoalbuminemia, could arise from inadequate intake, nutrient malabsorption, metabolic anomalies, or inflammatory processes. Depleted serum albumin levels might catalyze muscle atrophy, immunodeficiency, and accentuated malnutrition [29].

Mental health intricacies, particularly depression, have emerged as pivotal determinants in our predictive model. Through rigorous data analysis, we discerned a salient association between depressive states and amplified nutritional risk—a nexus that finds resonance with the seminal work of Kunugi et al. [30]. Individuals afflicted with depression often grapple with exacerbated negative affective states, a psychological milieu that invariably culminates in anorexic tendencies, thus leading to suboptimal caloric and essential nutrient intake. Persistently compromised dietary regimens predispose these individuals to multifarious metabolic dysregulations, amplifying the perturbations in their nutritional equilibrium. Our proposed model, in its essence, embodies an interdisciplinary paradigm, melding salient physiological, psychological, and biochemical indices tethered to nutritional precariousness. Each constituent element, though discrete, operates within a sophisticated, interconnected matrix, collectively modulating the patient's nutritional milieu. As clinicians and researchers, our purview necessitates a nuanced, multifactorial approach. Crafting bespoke nutritional interventions mandates a meticulous appraisal of this intricate interplay of determinants. By adopting such a granular, targeted approach, we can usher in interventions that are not only tailored but also potentiated for maximal efficacy, heralding enhanced patient prognostics and a marked elevation in their post-interventional quality of life.

### Generalizability and Clinical Utility of the Model

Leveraging frailty scores and utilizing LASSO regression for variable selection, this study constructed a linear predictive model to assess the nutritional risk in older stroke patients. The model's efficacy was substantiated through the analysis of the ROC curve, calibration curve, and clinical decision curve. The findings indicate that the developed Linear Prediction Model possesses commendable clinical discrimination, predictive accuracy, and practical utility.

Furthermore, this research incorporated external validation involving patients from diverse time periods. In the external validation cohort, the model exhibited consistent performance, suggesting notable generalizability. This predictive model equips clinical healthcare professionals with a practical tool for nutritional risk assessment. After brief training, they can use this model to forecast the nutritional risk in stroke patients, accounting for the comprehensive distribution of all delineated risk factors. This proactive approach facilitates early risk identification, enabling timely interventions. Such early actions can mitigate deteriorations in patients' quality of life and nutritional status during preliminary or post-onset therapeutic procedures.

## Limitations

First, this study employed a cross-sectional design, which inherently restricts the exploration of profound causal relationships. Future research could benefit from a longitudinal design, enabling continuous monitoring and dynamic observation of variations in patients' nutritional risks and associated variables. Second, while the model has undergone both internal and external validation, its applicability might still be susceptible to several influencing factors, including geographical variations, patient demographics, and the specificities of disease presentations. Lastly, despite the model demonstrating promising results during the validation process, it necessitates additional assessments to ascertain its efficacy and practicality within real-world clinical settings.

## Conclusions

The incidence of nutritional risk is notably elevated among frail older individuals who have suffered from strokes. This risk is intricately associated with various factors, including the capacity to perform daily activities (ADL), NIHSS scores, the presence of diabetes, BMI, grip strength, serum albumin, and depression. By establishing a predictive model anchored on frailty scores, healthcare professionals can more precisely pinpoint patients at heightened risk. Such identification paves the way for tailored nutritional intervention strategies, essential for enhancing nutritional status, facilitating rehabilitation, and ultimately augmenting the quality of life for these older stroke patients.

### Supplementary Information

Below is the link to the electronic supplementary material.

Supplementary file1 (DOCX 30 kb)

## Data Availability

The data generated during this study are subject to third-party restrictions and therefore are not publicly available. The data were used under license for the current study, and so are not accessible. Further inquiries can be directed to the corresponding author.
