# Supplementary material for: Development and Validation of a Nomogram for Predicting Nutritional Risk Based on Frailty Scores in Older Stroke Patients
Source: Aging Clin Exp Res. 2024 May 18;36(1):112. doi: 10.1007/s40520-023-02689-0 (PMC11102373; doi:10.1007/s40520-023-02689-0)

**Supplementary Materials**

**Supplementary Method S1 Detailed description of sample size calculation**

According to the clinical prediction model sample size estimation method is divided into 4 steps:

(1) Considering whether the sample size can accurately estimate the incidence of the outcome, the sample size was calculated by the incidence of the outcome event and the expected margin of error, assuming that the expected error is controlled within 5%, taking α = 0.05, and the sample was calculated to obtain a sample size of 385 cases.

$$n=\left( \frac{Z_{1-\alpha/2}}{\delta} \right)^{2}P\left( 1-P \right)=\left( \frac{1.96}{0.05} \right)^{2}0.5\left( 1-0.5 \right)\approx385$$

(i) n represents the sample content; (ii) δ represents the expected error, which takes the value of 0.05 in this study; (iii) P represents the value closest to 50% of the possible sample rate, which is taken as 50% if the overall picture is not clear; and (iv)$Z_{1-\alpha/2}$ takes the value of 1.96 was taken after consulting the table of Z-values.

(2) Ensuring that the new prediction model has a small prediction error for the target overall estimate, the estimated predictor variables in this study were 7, wanting a mean absolute error of no more than 0.05, a predicted incidence of debilitation of 50%, and a calculated sample size of 423 cases.

$$n=\exp\left( \frac{-0.508+0.259\ln\left( \varphi\right)+0.504\ln\left( p \right)-\ln\left( MAPE \right)}{0.544} \right)$$

$$n=exp(\frac{-0.508+0.259\ln\left( 0.5 \right)+0.504\ln\left( 7 \right)-\ln\left( 0.05 \right)}{0.544})\approx423$$

1. n is the sample size of the model development dataset; (ii) φ is the proportion of expected outcome events (≤0.5), which took the value of 0.5 in this study; (iii) P is the predictor variable data (≤30), which took the value of 7 in this study; and (iv) MAPE is the Mean Absolute Prediction Error, which took the value of 0.05 in this study.

(3) The minimum sample size required to reduce model overfitting ensured that the expected contraction rate was 10%, the estimated predictor variables were 7, the expected R^2^_CS_ was 0.2, and the calculated sample size was 398 cases.

(i) n is the sample size of the model development dataset; (ii) S is the expected shrinkage factor (≥0.9), which takes the value of 0.9; (iii) P is the number of predictor variables (≤30), which takes the value of 7 in this study; and (iv) R^2^_CS_ is the index for evaluating the noise ratio of the model, which takes the value of 0.2 in this study.

(3) Calculating the sample size that guarantees a small difference between the developed model and the optimally adjusted values, it is known from the literature that maxR^2^_CS_ takes a value of 0.75 when R^2^_CS_ takes a value of 0.2, and the sample size is calculated to be 378 cases.

$$S=\frac{R_{cs}^{2}}{R_{cs}^{2}+\delta max(R_{cs}^{2})}=\frac{0.2}{0.2+0.05\times0.75}=0.842$$

$$n=\frac{p}{(S-1)\ln(1-\frac{R_{cs}^{2}}{S})}=\frac{7}{(0.842-1)\ln(1-\frac{0.2}{0.842})}\approx164$$

Combining the above 4 steps, the modeling set sample size of 423 cases is needed in this study. According to the principle that the sample size for external validation of risk prediction models is generally 1/4 to 1/2 of the sample size of the modeling set, and considering the 10% lost visit rate, the sample size of the validation set should be 116~133 cases.

| **Supplementary Table S1 General characteristics of the external validation set**  Table S1 Comparison of general information of patients with different nutritional risk subgroups in the external validation set | | | | |
| --- | --- | --- | --- | --- |
| Characteristics | Nutritional risk individuals（n=219） | Non-nutritional risk individuals（n=215） | χ^2^/Z值 | *P*值 |
| Age [year, case (%)] |  |  | -4.275 | ＜0.001 |
| 60~70 | 19（27.1） | 43（65.2） |  |  |
| 70~80 | 33（47.1） | 16（24.2） |  |  |
| 80~90 | 18（25.7） | 7（10.6） |  |  |
| Gender [case (%)] |  |  | 3.062 | 0.080 |
| Male | 48（64.9） | 31（50.0） |  |  |
| Female | 26（35.1） | 31（50.0） |  |  |
| ADL [case (%)] |  |  | 48.084 | ＜0.001 |
| independent. | 11（15.7） | 44（66.7） |  |  |
| mildly dependent | 18（25.7） | 17（25.8） |  |  |
| Moderate dependent | 27（38.6） | 4（6.1） |  |  |
| heavy dependent | 14（20.9） | 1（1.5） |  |  |
| NIHSS score [case (%)] |  |  | 14.174 | ＜0.001 |
| 0~1 | 22（31.4） | 42（63.6） |  |  |
| 1~4 | 33（47.1） | 17（25.8） |  |  |
| 5~15 | 15（21.4） | 7（10.6） |  |  |
| Diabetes [case (%)] | 40（57.1） | 21（31.8） | 8.808 | 0.003 |
| calf circumference＜31 [cm, case (%)] | 53（75.7） | 16（24.2） | 36.008 | ＜0.001 |
| Albumin [g/dL, M (25%, 75%)] | 119（108, 134） | 135（124, 147） | -6.780 | ＜0.001 |
| total protein [g/dL, M (25%, 75%)] | 35.89（33.7, 39） | 43.1（40.68, 45.48） | -0.048 | ＜0.001 |
| depression [case (%)] | 32（45.7） | 8（12.1） | 26.982 | ＜0.001 |

**Supplementary Figure S1 Importance ranking of variables**


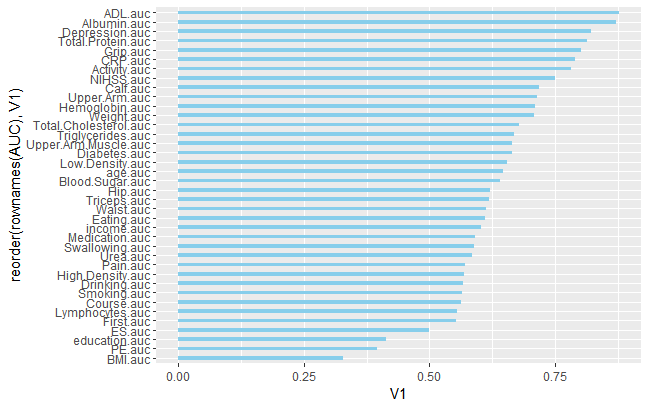

Supplement: Supplementary file 1 — Supplementary file1 (DOCX 30 kb) [file 40520_2023_2689_MOESM1_ESM.docx]
